# *Brucella melitensis*, a latent “travel bacterium,” continual spread and expansion from Northern to Southern China and its relationship to worldwide lineages

**DOI:** 10.1080/22221751.2020.1788995

**Published:** 2020-07-14

**Authors:** Xiong Zhu, Zhongzhi Zhao, Shuyi Ma, Zhiwei Guo, Miao Wang, Zhenjun Li, Zhiguo Liu

**Affiliations:** a Sanya People’s Hospital, Sanya, People’s Republic of China; b State Key Laboratory for Infectious Disease Prevention and Control, National Institute for Communicable Disease Control and Prevention, Chinese Center for Disease Control and Prevention, Beijing, People’s Republic of China; c School of Medical Technology, Baotou Medical College, Baotou, People’s Republic of China; d Qinghai Institute for Endemic Diseases Prevention and Control, Xining, People’s Republic of China; e Inner Mongolia Autonomous Region Center for Comprehensive Disease Control and Prevention, Huhhot, People’s Republic of China; f Ulanqab Centre for Endemic Disease Prevention and Control, Jining, People’s Republic of China

**Keywords:** *Brucella melitensis*, species/biovars, genetic relatedness, MLVA, WGS-SNP, China

## Abstract

Brucellosis caused by *Brucella melitensis* is considered to be one of the most important zoonotic diseases in China. In this study, Conventional bio-typing, MLVA (multiple locus variable-number tandem repeat analysis), and WGS (whole-genome sequencing)-SNP (single nucleotide polymorphism) were used to study the genetic similarity of *B. melitensis* in northern and southern China and analyze its relationship with worldwide lineages. Currently, the distribution of species/biovars of *B. melitensis* has obviously changed, and *B. melitensis* has become the dominant species in southern regions of China. Strains from the southern had a common geographic origin with strains from the northern. Many MLVA-16 events were shared in the genotypes of the southern and northern strains, suggest that genotypic movement occurred from north to south. Based on WGS-SNP analysis, strains from different provinces were closely related and may have descended from one common ancestor, suggests that the southern strains originated from northern China. These data indicate that *B. melitensis* is a latent “travel bacterium” that spread and expanded from North China to South China. Moreover, *B. melitensis* strains from China are also genetically related to strains from other Asian regions (Kazakhstan, Russia, Mongolia, and India). The movement of infected sheep and their products requires control.

## Introduction

Brucellosis, a common zoonotic disease globally, is caused by bacteria of the genus *Brucella* [1], which are nonmotile, gram-negative α-proteobacteria that are facultative intracellular pathogens [2]. Human brucellosis is largely dependent on animal reservoirs and through direct contact with infected animals or consumption of contaminated animal products [3]. At present, *Brucella. melitensis*, *Brucella. abortus*, and *Brucella. suis* remain the main causes for human and animal brucellosis worldwide [4]. Chronic infections with severe complications in humans are a major public health problem [5,6]. The brucellosis epidemiological situation remains complex, and there are serious epidemics in many low-income countries, including in the Mediterranean region, South and Central America, Africa, Asia, the Arabian Peninsula, the Indian subcontinent, Eastern Europe, the Middle East, and China [7]. Despite its low mortality rates, brucellosis is a very important public health problem in rural and pasturing areas in China [8]. Brucellosis has reemerged since 1995, and human brucellosis has been reported in all mainland provinces, a total of 513,034 brucellosis cases were recorded from 1955 to 2014, of which 99.3% were in northern China [9]. Animal husbandry suffers great economic losses from brucellosis due to reduced productivity, the culling of livestock, and costs of associated control measures [10,11]. The incidence of human brucellosis in southern China increased in 2005 and 2014 and the affected area expanded from northern to southern coastal and southwestern areas [9,12]. *B. melitensis* is responsible for the vast majority of brucellosis in humans and animals in China [13], but the genetic relatedness of *B. melitensis* within China and its relationship to strains in other world areas is unknown. Investigation of species and genotype distributions, genetic relatedness, and molecular epidemiology of the main circulating strains is essential for understanding the epidemiology of human brucellosis, managing disease outbreaks and for establishing efficient prevention and control programmes [14].

Multiple-locus variable-number tandem-repeat analysis (MLVA) has high power to discriminate closely related strains and can be used for tracing infections [15], achieves result largely in agreement with WGS-SNP-based typing [16]. In addition, its low cost and fast results allow its use as a routine first-line assay [17]. Ma *et al*. [18] reported that the *Brucella* strain in Qinghai was different from strains in other regions of the world, possibly owing to the unique geography, such as the high altitude, of the QTP. Extensive genotype-sharing events between isolates obtained from humans and animals showed that yaks, sheep, and blue sheep were important zoonotic reservoirs of brucellosis that caused human infections. Liu *et al.* [13] reported that human brucellosis in Ulanqab, Inner Mongolia (China) occurred as a multipoint outbreak epidemic caused by multiple common sources of infection. Many shared MLVA-16 genotypes were observed among isolates from different regions of Ulanqab and from other provinces of China. This suggests that infected animal movement between different regions is not controlled. Consequently, an investigation of the genetic relatedness, molecular epidemiology, and potential transmission route of *B. melitensis* from humans and animals over the whole country is needed. The purpose of this study was to determine the distribution profiles, genetic relatedness, and potential transmission pattern of 1,382 *B. melitensis* collected from 29 different regions from humans and animals at the whole-country scale.

## Materials and methods

### Ethics statement

This study was carried out according to the principles of the Declaration of Helsinki. This study is a retrospective investigation of historical strain collections using molecular typing methods, and the research protocol was approved by the Ethics Committees of the National Institute for Communicable Disease Control and Prevention and the Chinese Center for Disease Control and Prevention. All strains from humans were collected as a part of a standard clinical investigation of patients with suspected brucellosis. The patients were anonymized. All strains from animals were obtained during related research on animal brucellosis. The majority *Brucella* strains (human and animals) used in this study were collected from published academic articles found on PubMed and Chinese life science databases (e.g. WanFang data and CNKI) and MLVA bank (http://microbesgenotyping.i2bc.paris-saclay.fr/databases).

### Clinical strains characterization

A total of 1382 *B. melitensis* (385 in animals and 997 in humans) were collected from patients and animals from 1955 to 2018 in 29 provinces of China. Fewer *B. melitensis* strains from Anhui Provinces and Tibet have been reported but MLVA genotyping of these strains has not yet completed. Because of this, strains from these regions were excluded from this study. All strains were isolated and identified according to standard bacteriology approaches [19]. Biotypes were assigned by conventional identification methods, and all strains were gram negative, agglutinated with polyvalent brucellosis serum, had oxidase and catalase activity, did not produce H_2_S, synthesized urease, and were capable of growing in atmospheric conditions. Both AMOS-PCR [20] and ladder PCR [4] were applied to verify the results from bio-typing assays.

### DNA preparation, genotyping, and data analysis

Bacterial cultures were scrapped from the surfaces of solid agar medium. DNA was isolated using the QIAamp DNA Mini Kit (Qiagen, United States) according to the manufacturer’s instructions. The MLVA-16 assay was performed as previously described [21,22]. Briefly, 16 loci were divided into three panels: panel 1 (also called MLVA8), panel 2A, and panel 2B. The combination of panels MLVA8 and 2A was called MLVA11, while the combination of all three panels (16 loci) was designated MLVA16. The MLVA11 panel allows for tracing the geographic origin of strains analyzed, while the panel 2B loci are highly discriminatory and their combination with MLVA11 was used in tracking local outbreaks. PCR was used to determine the number of repeats from a sample, and its products were purified and directly sequenced using an ABI Prism Big Dye Terminator. Size analysis of VNTR repeats was performed using GeneMapper 4.1 (Applied Biosystems). Dendrograms from strains analyzed (Table S1) were constructed using BioNumerics 5.0 (Applied Maths, Sint-Martens-Latem, Belgium) based on the categorical coefficient and unweighted pair group method using arithmetic averages (UPGMA). Minimum spanning trees (MST) were constructed based on MLVA-11 (Table S2) and MLVA-16 (Table S3) data using BioNumerics 7.6 to investigate the geographic origin and genetic relatedness of strains. Phylogenetic analysis of representative strains (Table S4) was performed based on WGS-SNP using the maximum parsimony method [17], *B. abortus* bv.1 str. 9–941 used as the outgroup strain. Microsoft Excel 2016 (Microsoft, Redmond, WA, USA) was used for data processing, and ArcGis 10.5 (ESRI, Redlands, CA, USA) was applied to display analysis results.

## Results

### Distribution characteristics of species biotypes of Chinese *B. melitensis* strains

**Figure 1.**
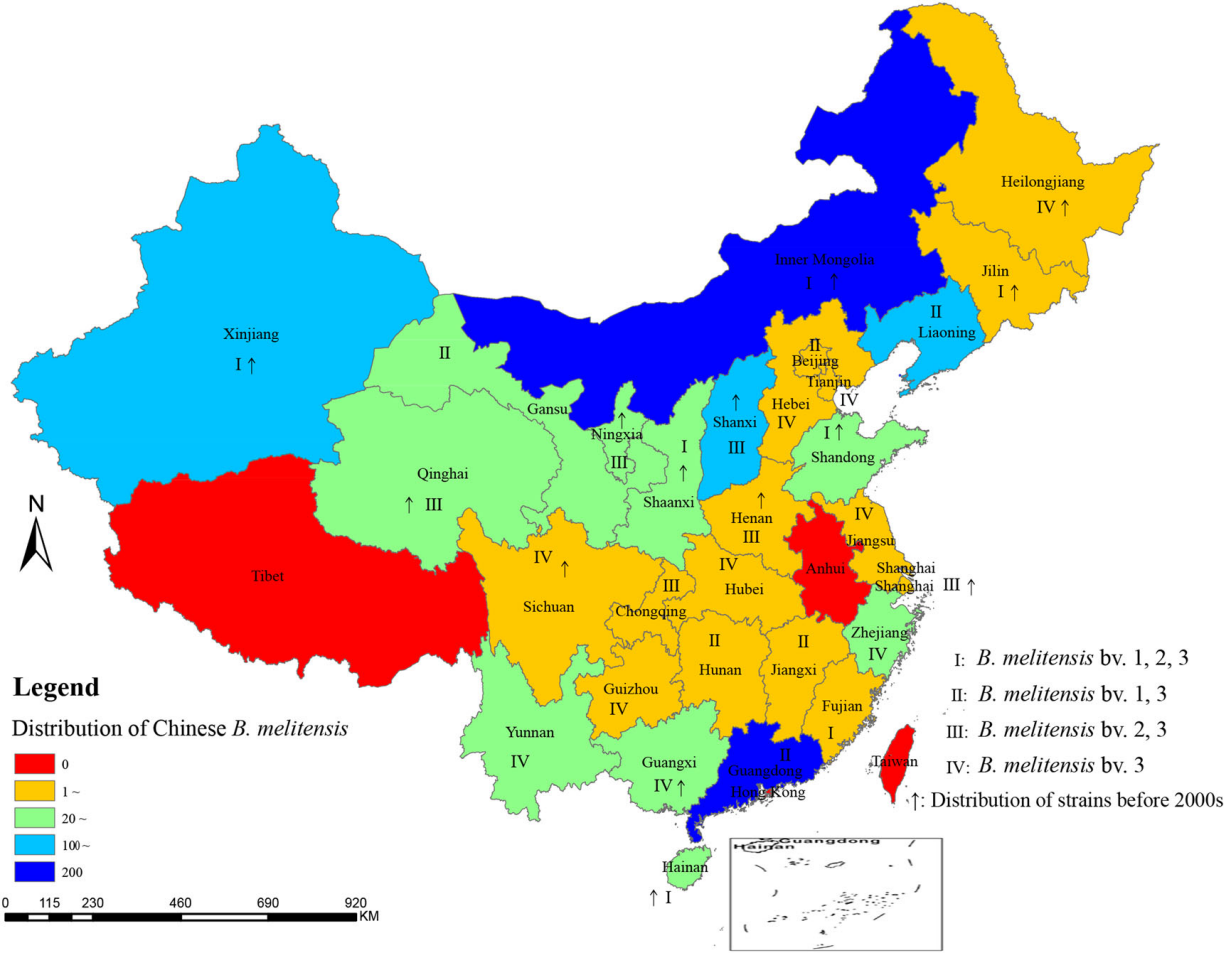
Geographic distribution of *B. melitensis* samples in China. Note: the map of this study does not represent the true borders of administrative regions of China.

A total of 1382 *B. melitensis* strains were collected from 29 provinces (including autonomous regions and cities); with the exceptions of Tibet and Anhui, all mainland provinces had obtained *B. melitensis* strains. Among them, 977 were obtained from human blood samples and 385 strains were recovered from animal samples: 291 in Inner Mongolia, 200 in Guangdong, 165 in Liaoning, 113 in Xinjiang, 107 in Shanxi, 91 in Qinghai, 54 in Shandong, 52 in Ningxia, and other regions contained 3–44 strains as shown in Table 1. A total of 35.5% of the strains were from Inner Mongolia and Guangdong Provinces; the former is a historical area for brucellosis, and the latter is an emerging region for brucellosis. 1382 *B. melitensis* samples were divided into four distinct epidemic areas: seven provinces in which all three *B. melitensis* biovars (1, 2, and 3) were collected (I); six provinces in which *B. melitensis* biovars bv. 1 and 3 were found (II); six provinces in which *B. melitensis* bv. 2 and 3 were found (III); and ten provinces in which *B. melitensis* bv. 3 was obtained (IV) (Figure 1). *B. melitensis* strains were found in 14 provinces in China before 2000, while strains were found in 29 provinces in 2018 (Figure 1). These data demonstrated that *B. melitensis* has expanded its distribution to all of mainland China.

**Table 1.** Location, numbers, percentages (%), species, and hosts of 1382 *B. melitensis* isolates in 29 provinces.

Province	No.	%	Species-biovar	Host
Hubei	3	0.22	*B. melitensis* bv. 3	Human
Tianjin	3	0.22	*B. melitensis* bv. 3	Human
Beijing	4	0.29	*B. melitensis* bv. 1, 3	Human
Shanghai	4	0.29	*B. melitensis* bv. 2, 3	Human, Sheep, Cattle
Chongqing	4	0.29	*B. melitensis* bv.2, 3	Human, Sheep
Guizhou	5	0.36	*B. melitensis* bv. 3	Human, Goat
Hunan	5	0.36	*B. melitensis* bv. 1, 3	Human
Sichuan	7	0.51	*B. melitensis* bv. 3	Human Sheep, Cattle, Yak
Heilongjiang	8	0.58	*B. melitensis* bv. 3	Human, Sheep
Jilin	8	0.58	*B. melitensis* bv. 1, 2, 3	Human, Sheep, Cattle, Deer
Henan	12	0.87	*B. melitensis* bv. 2, 3	Human, Sheep
Jiangxi	14	1.01	*B. melitensis* bv. 1, 3	Human
Jiangsu	17	1.23	*B. melitensis* bv. 3	Human
Fujian	18	1.30	*B. melitensis* bv. 1, 2, 3	Human
Hebei	19	1.37	*B. melitensis* bv. 3	Human, Sheep
Yunnan	20	1.45	*B. melitensis* bv. 3	Human
Guangxi	22	1.59	*B. melitensis* bv. 3	Human
Gansu	26	1.88	*B. melitensis* bv. 1, 3	Sheep
Shaanxi	28	2.03	*B. melitensis* bv. 1, 2, 3	Human
Zhejiang	38	2.75	*B. melitensis* bv. 3	Human, Goat
Hainan	44	3.18	*B. melitensis* bv. 1, 2, 3	Human, Sheep
Ningxia	52	3.76	*B. melitensis* bv. 2, 3	Human, Sheep, Goat
Shandong	54	3.91	*B. melitensis* bv. 1, 2, 3	Human, Sheep
Qinghai	91	6.58	*B. melitensis* bv. 2, 3	Human, Sheep, Cattle, Blue sheep, Yak, *Pseudois nayaur*, Tibetan gazelle
Shanxi	107	7.74	*B. melitensis* bv. 2, 3	Human, Sheep, Cattle
Xinjiang	113	8.18	*B. melitensis* bv. 1, 2, 3	Human, Sheep, Cattle, Goat, Yak
Liaoning	165	11.94	*B. melitensis* bv. 1, 3	Human
Guangdong	200	14.47	*B. melitensis* bv. 1, 3	Human
Inner Mongolia	291	21.06	*B. melitensis* bv. 1, 2, 3	Human, Sheep, Cattle, Camel

### Geographic origins of Chinese *B. melitensis* strains based on MLVA-11

**Figure 2.**
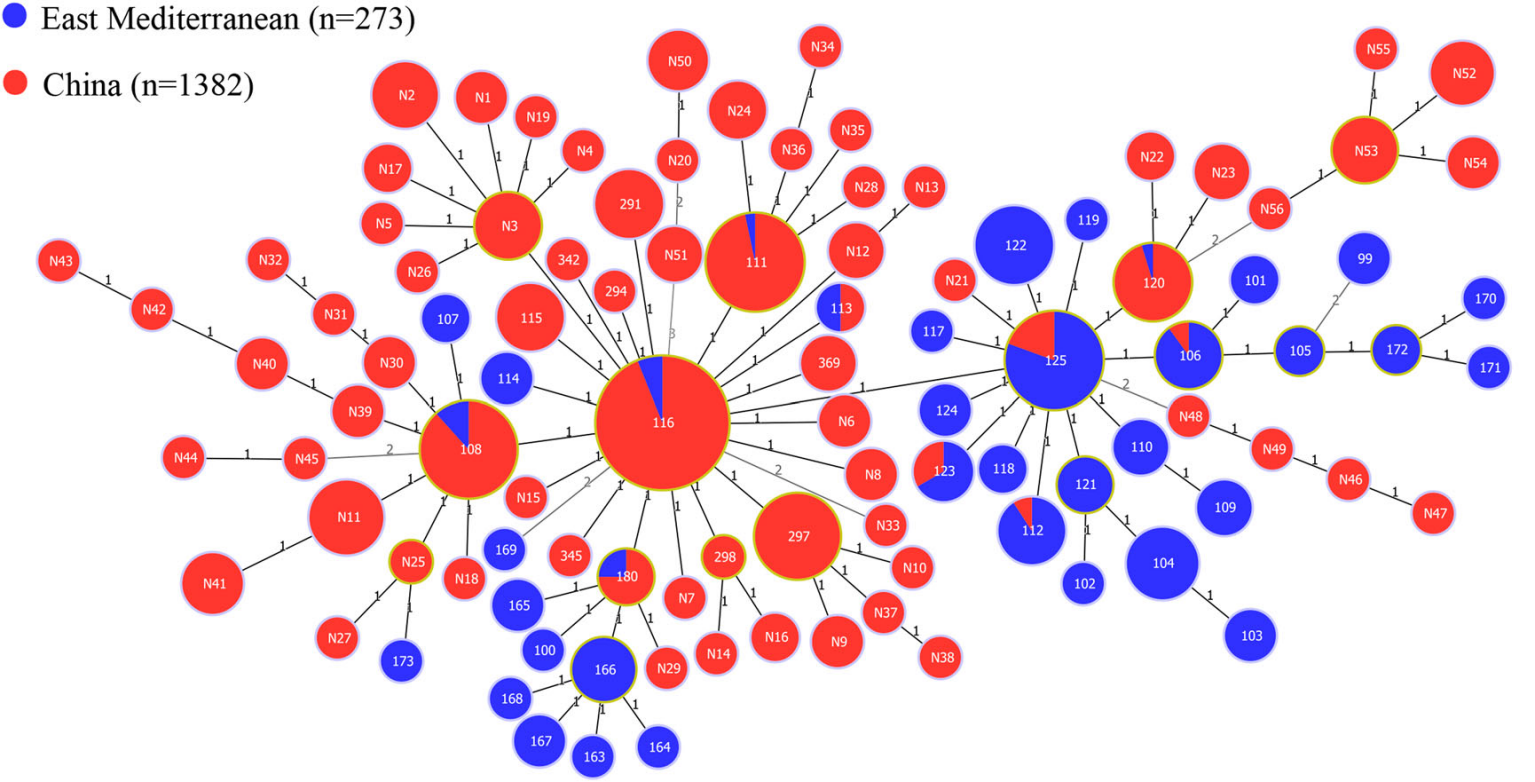
Minimum spanning tree for *B. melitensis* using MLVA-11 data with Chinese isolates (red) and East Mediterranean isolates (blue). Note: numbers in lines show the values of locus variants and numbers in nodes represent MLVA-11 genotypes.

**Figure 3.**
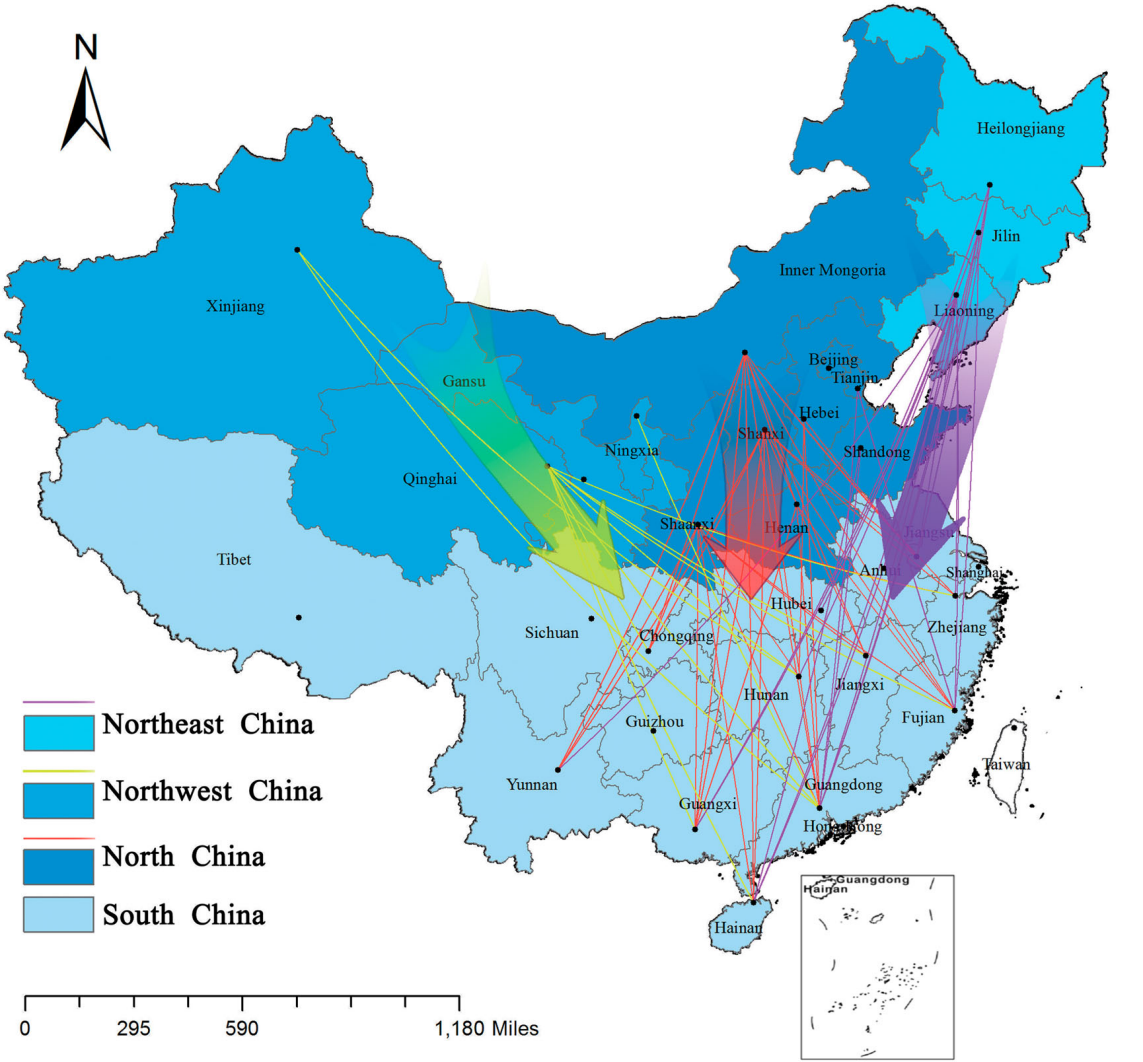
Transmission pattern of *B. melitensis* isolates from humans. Note: the map of this study does not represent the true borders of administrative regions of China.

**Figure 4.**
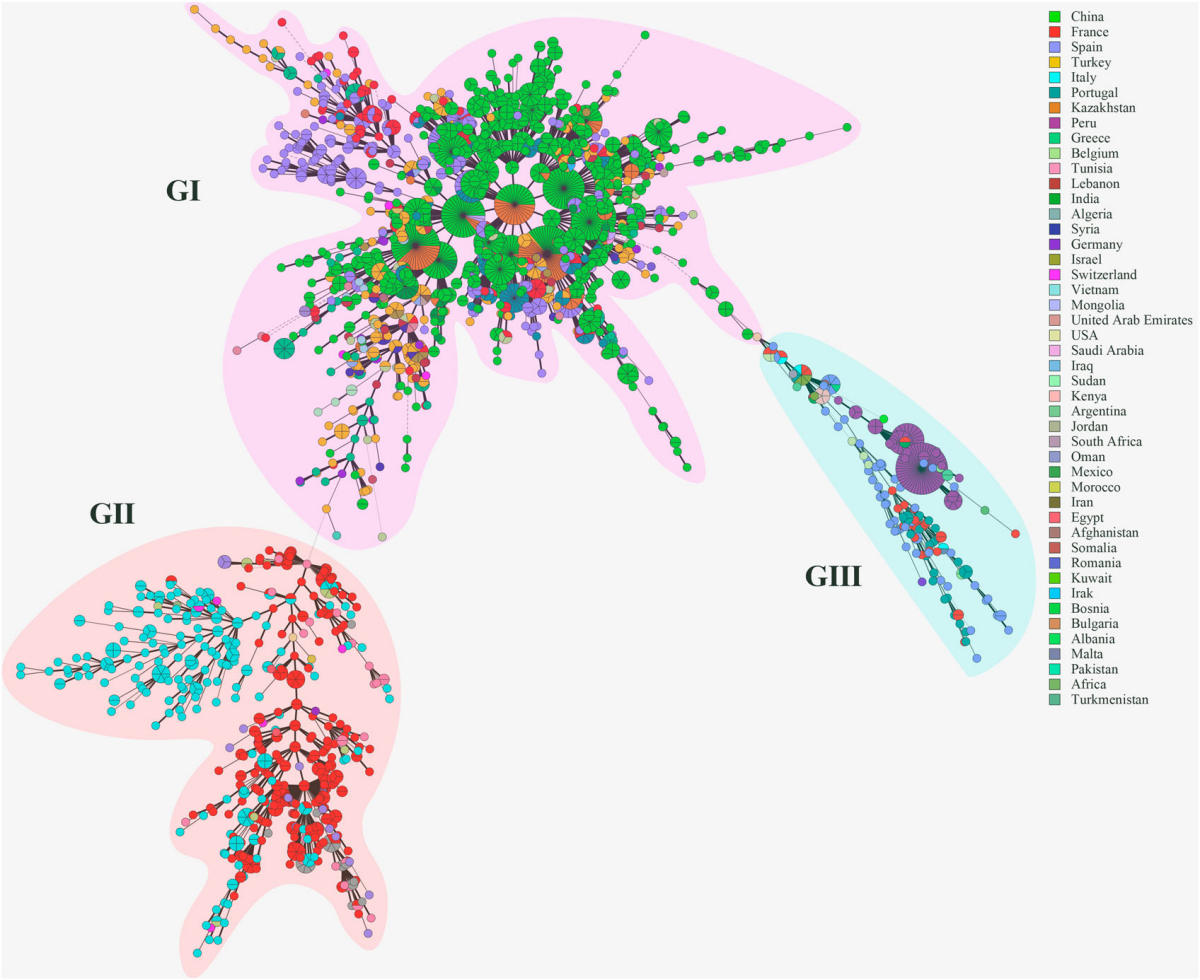
Minimum spanning tree (MST) was constructed using MLVA-16 data on a global scale.

1382 *B. melitensis* strains yielded 71 MLVA-11 genotypes, including 53 new MLVA-11 genotypes and 18 known genotypes, of which 69 belonged to the East Mediterranean lineage, and two new (N50 and N20) genotypes were of the Americas lineage (Figure 2). Seven MLVA-11 genotypes (116, 111,108, 297, N11, N24, and N3) made up the predominant circulating genotypes, of which 69% (951/1382) were genotype 116, which was shared by strains from 28 different provinces in northern and southern China (S. Figure 1). These southern provinces had fewer *B. melitensis* before 2000 (S. Figure 1), suggesting that there has been continuous expansion from northern to southern regions. These dominant MLVA-11 genotypes were shared by strains from 5 to 28 distinct provinces (S. Figure 2), of which 89 strains were MLVA-11 genotype 111, accounting for 6.4% (89/1382) and distributed in eleven provinces; 76 strains were MLVA-11 genotype 108, accounting for 5.5% (76/1382) and distributed in seven regions; 39 strains were MLVA-11 genotype 297, accounting for 2.8% (39/1382) and shared by strains from ten provinces; 20 strains were MLVA-11 genotype 120, accounting for 1.4% (20/1382) and shared by strains from five regions, dominating in southern regions including Fujian, Hainan, Yunnan, and Guangxi provinces. The distributed range in the remaining genotypes was limited. All predominant MLVA-11 genotypes were shared by strains from different epidemic periods of brucellosis, including 1950–1970, 1980–2000, and 2001–2018 (S. Figure 3). Moreover, 14 circulating MLVA-11 genotypes were also shared by strains from north and south China (S. Figure 4).

### Epidemiological characteristics of animal and human brucellosis

**Figure 5.**
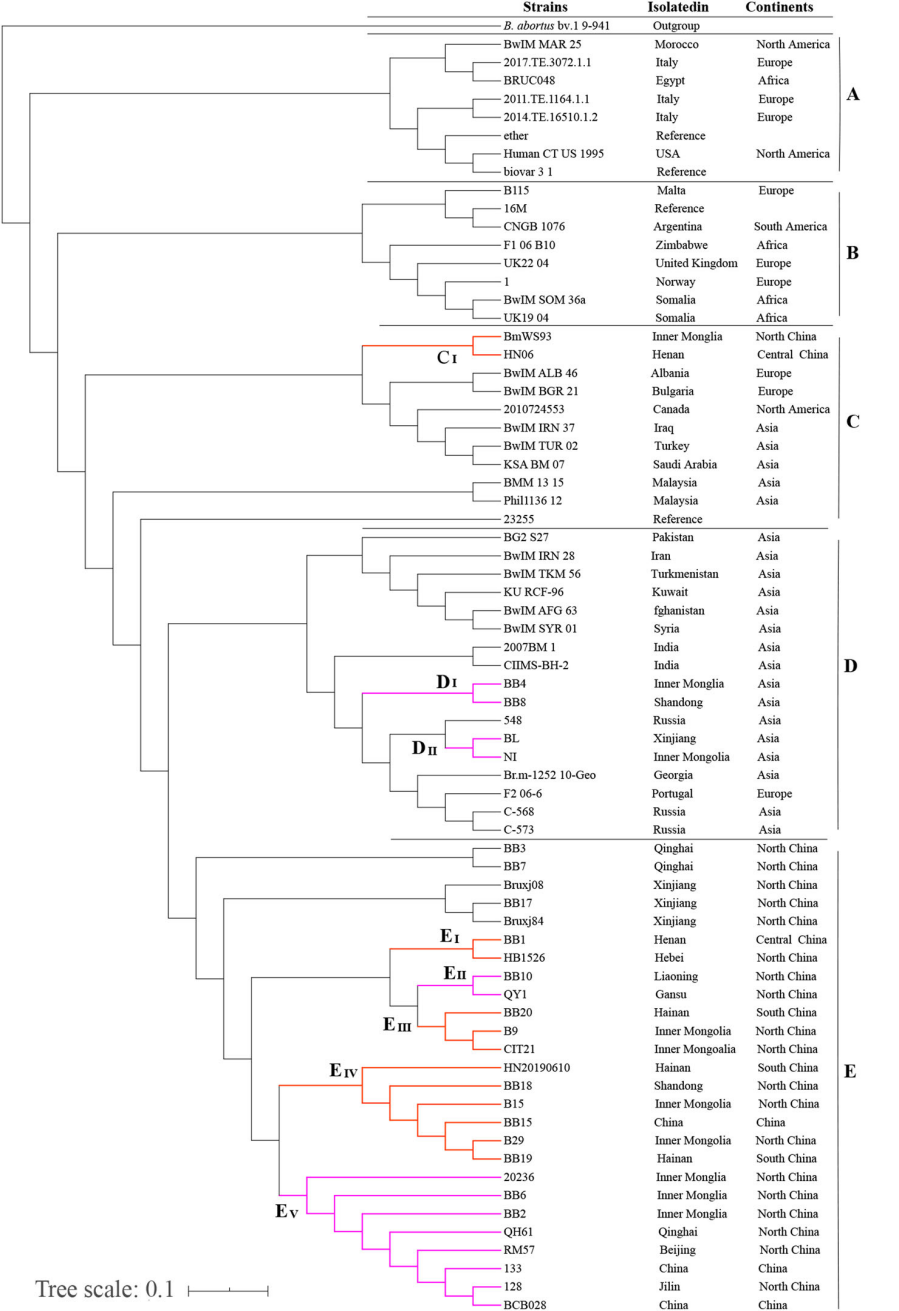
Phylogenetic tree of *B. melitensis* strains based on WGS-SNP over all of global. (Clades coloured with orange that representation strains from South were closely related to North and Central regions, Clades coloured with purple showed that there were closely related among strains from North regions.)

Based on the MLVA-16 genotype, 385 animal *B. melitensis* strains were sorted into 157 genotypes, of which 91 were shared genotypes in that each genotype present in 2–17 strains, and the cluster rate of strains was 82.3% (317/385). Among the 91 shared genotypes, 24 shared genotypes were present in 103 strains from two to four different provinces (S. Figure 5) (Table S5), accounting for 32.5% (103/317); the other shared genotypes were all from strains from the same provinces. A total of 977 human *B. melitensis* strains were divided into 391 MLVA-16 genotypes, of which 158 shared genotypes were present in 744 strains, and the cluster rate of these strains was 76.2% (744/977); 88 genotypes were in 268 strains from two to eight different provinces (Table S5), accounting for 36.0% (268/744); and the remaining 233 strains represented single genotypes, with each being an independent strain, accounting for 24% (233/977). The strains from the southern provinces had more similar MLVA-16 genotypes with strains from northern regions, the latter being a historical area of animal and human brucellosis (Figure 3). In particular, three shared genotypes were present in strains from both southern and northern provinces, including Jilin, Qinghai, Guangdong, Inner Mongolia, Guangxi, Fujian, Liaoning, and Shaanxi; Inner Mongolia, Qinghai, Henan, Guangdong, Shanxi, Shaanxi, and Guangxi; and Inner Mongolia, Jilin, Shanxi, Liaoning, Shandong, Guangdong, and Hainan (S. Figure 6). The other 70 shared genotypes were present in strains from the same provinces, accounting for 64% (476/744). Meanwhile, completely identical MLVA-16 genotypes were shared by strains from the three different epidemic periods (S. Figure 7). Moreover, many shared genotypes were observed among strains from livestock, humans, and wild animals (S. Figure 8).

### Genetic relatedness of *B. melitensis* strains on a global scale

To reveal the genetic links of Chinese strains with those in the rest of the world, the genetic relatedness among 3480 *B. melitensis* strains on a global level were compared using MST based on MLVA-16 data. MST analysis showed that these strains sort into three groups (G I ∼ III) (Figure 4). Many shared genotypes were observed in strains from this study with strains from Kazakhstan, Turkey, and Mongolia (Figure 4, G I); these regions are important states along the silk road with close geographies. However, there were significant genetic differences between strains from China and Italy, France, and Peru (Figure 4, G II and III). Phylogenetic analysis of strains was performed based on WGS-SNP of 74 *B. melitensis* strains from NCBI GenBank. Phylogenetic analysis based on whole-genome SNPs and geographical distribution of the isolates revealed spatial clustering of the *B. melitensis* isolates were divided into five clades (A-E) (Figure 5). The Mediterranean strains, identified as clade A, occupied the basal node of the phylogenetic tree. The majority of the Chinese *B. melitensis* strains clustered into clade E and represented the Asia lineage (Figure 5). Strains obtained from southern and central regions had close relationships with strains from northern regions, China, including the Inner Mongolian Autonomous Region, Xinjiang, and Shandong Provinces (Figure 5 C (I), D (I and II), E (I - V)), which are ongoing epidemic regions of animal and human brucellosis. However, *B. melitensis* strains from Chinese provinces had a close genetic relationship to strains from other Asian regions including Russia, India, and Georgia, which are traditional epidemic regions of brucellosis (Figure 5(C and D)).

## Discussion

Brucellosis is the most common zoonotic disease and it is mainly caused by *B. melitensis* infection (biovars 1 and 3). Brucellosis poses a threat to both animals and humans [23]. A previous study showed that 84.5% of the *Brucella* strains isolated from humans with brucellosis in China were *B. melitensis* [24]. *B. melitensis* strains are now widely distributed throughout China and the distribution of pathogenic species of brucellosis in China has obviously changed. Since the 1950s, *B. melitensis* was most common in the grassland areas of northern China, where sheep and goats are the main livestock [24]. *B. melitensis* strains were found in 14 provinces in China before 2000. Now, *B. melitensis* strains occur in 29 provinces (autonomous regions and cities) and *B. melitensis* is the dominant circulating species in southern regions of China [25,26]. This supports our hypothesis that *B. melitensis* has spread and expanded from northern to southern China. Brucellosis occurred earlier in the north than in the south and due to the introduction of northern sheep and other species, human brucellosis is increasing in the southern provinces [12]. Since 2010, human brucellosis has occurred or reappeared in all provinces in southern China [27]. These data confirm that the geographic distribution of the disease has also evolved, with the movement of *B. melitensis* strains to the south.

In this study, 69% (951/1382) of the strains were genotype 116 and belonged to the East Mediterranean lineage. Genotype 116 is shared by strains from 28 different provinces in northern and southern China, revealing that these strains had a common geographic origin. Genotype 116 is responsible for the vast majority of *Brucella* infections in humans and animals [28] and is predominant in many countries, accounting for more than 77% of cases in Portugal and Kazakhstan, 37% in Spain, 16% in Turkey, and 10% in France [29]. Many shared MLVA-16 genotypes were observed among strains from northern and southern regions, and three brucellosis epidemic periods. This finding coincides with species distributions and geographic origin profiles of strains in this study. These data suggest that *B. melitensis* spreads and expands continually and has moved from northern to southern, China [30], while affected area now covers all of mainland, China [9].

Brucellosis is associated with large-scale farming and trading of sheep and goats. The inventory of sheep is correlated with the presence of brucellosis cases in mainland China [31]. The non-regulated animal trade has an important impact on the dissemination of brucellosis. Infected sheep and their products that have not been quarantined in the north before import to the south may be the main cause for the increasing incidence of human brucellosis in southern China [27]. Introduced infected sheep from northern regions may have led to a brucellosis outbreak epidemic in Zhejiang Province, China [32]. The increasing demand for meat and expanding animal husbandry in southern China have also increased the infection risk for brucellosis due to occupational exposure [33]. Brucellosis is an emerging disease and there is unfamiliarity with its infection in most provinces in southern China. Therefore, the occupational protections used in populations are relatively few. Control of brucellosis in southern China requires strict restrictions on the inter-provincial movement of infected animals. Livestock serologically negative for brucellosis can be allowed to move without restriction. Livestock that test positive for brucellosis, or those with unknown disease status, should only be allowed to move if they have a negative serological test issued up to 30 days before movement [34]. An improved culling policy is also needed. Sick animals cannot be properly disposed because of unacceptable compensation funds for farmers and this leads to persistence of the source of infection [35]. There should be improved capability in immunization, quarantine, diagnosis, and treatment in animal disease control and prevention organizations to meet the increasing need for disease control. Lastly, improved awareness of the need for personal protection for those working with animals or animal products is needed. Individuals should wear gloves and other appropriate protective clothing.

Many shared genotypes were observed among strains from different hosts. These data showed a potential transmission pattern for *B. melitensis* in China and demonstrate direct or indirect transmission among livestock and wild animals, eventually infecting humans. Previous reports have shown that wild animals are a significant brucellosis reservoir for livestock and humans [36,37]. To better understand the epidemiological characteristics of brucellosis in China, studies on brucellosis epidemics in wild animals at the country level are needed.

MST analysis showed that the strains studied here had a common geographic origin and a close relationship with strains from Kazakhstan, Mongolia and Turkey [15]. The most MLVA-16 shared genotypes were found for strains from China and Kazakhstan. These regions are geographically close and have historically exchanged livestock. Animals were a common form of payment in ancient commerce, and the modern version of this practice has promoted disease transmission [38].

Based on WGS-SNP analysis, the Mediterranean strains, identified as clade A, occupied the basal node of the phylogenetic tree. This indicates that *B. melitensis* may have originated in the Mediterranean regions. Brucellosis may have been identified in the late Roman era; however, the disease was first described by Sir David Bruce, Hughes, and Zammit while working in Malta [39]. Clade E comprised 26 out of the 74 *B. melitensis* strains used in the study. It represented the largest *B. melitensis* genotype, with isolates collected from diverse locations of China. It exhibited a ladder-like phylogram, suggesting a possible single introduction of these genotype strains into China [40]. WGS-SNP analysis showed close relationships among strains from southern provinces, central, and many northern regions, indicating that *B. melitensis* strains from the southern region originated from northern regions. This conclusion is consistent with previous reports that the sources of infection of human brucellosis in southern regions (Guangxi, Hunan and Hainan Provinces) originated in northern provinces, including Inner Mongolia [25,26,41]. However, *B. melitensis* strains from China have a genetic relationship to strains from Asian regions including Russia, India, and Georgia, which are traditional epidemic regions of brucellosis. Although few *B. melitensis* isolates were from Russia, they are genetically similar to Chinese strains [42], and these strains had high homogeneity [43]. India harbours the largest ruminant populations and there are high seroprevalence estimates of brucellosis in livestock and humans [44]. Importantly, the absence of a clear differentiation according to territorial affiliation between these regions indicates the frequent penetration of the *B. melitensis* strains from one country to another [43], and active trade based on the ancient Silk Road, Tea Horse Road and Trans-Eurasia exchange among these nearby regions could have promoted this process. Brucellosis has a “knows no borders” character, making it challenging to monitor and control [45]. A positive response to a “National brucellosis control plan (2016–2020)” is needed. It is also necessary to enforce animal controls, vaccinate all susceptible animals, and increase the level of education and awareness among people, especially regarding contact with infected animals and consumption of contaminated milk and other byproducts.

This study has several limitations. First, there was considerable variability in the number of strains collected among different regions and periods. In some high-endemic areas of brucellosis, Jilin and Heilongjiang had fewer strains, affecting the study conclusions. Second, data on the distribution of prevalence of animal brucellosis, sheep population mobility, and strains obtained from animals (sheep) from southern provinces were lacking. Further animal studies including seroprevalence and strain distributions are essential. Genomic data of strains from China in the Gene bank were limited and additional phylogenetic analysis of more Chinese *B. melitensis* is warranted.

## Conclusion

Although a national brucellosis control programme in China has been ongoing for many years (2009–2020), disease prevalence has not obviously declined. This indicates a need to reformulate the existing management strategies. At present, *B. melitensis* is the predominant species in southern China. Sheep and animal products are traded frequently between northern and southern regions and this promotes the spread and expansion of *B. melitensis* strains. Because sheep play a large role of the spread of brucellosis, surveillance and disease countermeasures for sheep populations should be a priority. Quarantine and inspections of sheep transfer and trade are urgently needed. Brucellosis is a disease that has spread across extensive regions and provinces and is transmitted by food, air, and soil. It occurs in areas dominated by traditional hygiene practices and these areas have limited access to health services. We encourage government organizations (veterinary and health authorities) to play a greater role in disease management. The active participation of livestock producers as well as industry partners is also essential.

## Supplementary Material

Table_S5.xlsx

Table_S4.xlsx

Table_S3.xlsx

Table_S2.xlsx

Table_S1.xlsx

S._Fig._8.tif

S._Fig._7.tif

S._Fig._6.tif

S._Fig._5.tif

S._Fig._4.tif

S._Fig._3.tif

S._Fig._2.tif

S._Fig._1.tif

## Data Availability

All data generated or analyzed during this study are included in this published article and its supplementary information files.

## Abbreviations

**Abbreviations**: MLVA: multiple locus variable-number tandem repeat analysis; WGS: whole genome sequencing; SNP: single nucleotide polymorphism; MST: minimum spanning tree
