# Supplementary figures and images for: Brucella melitensis, a latent “travel bacterium,” continual spread and expansion from Northern to Southern China and its relationship to worldwide lineages
Source: Emerg Microbes Infect. 2020 Jul 14;9(1):1618–27. doi: 10.1080/22221751.2020.1788995 (PMC7473006; doi:10.1080/22221751.2020.1788995)

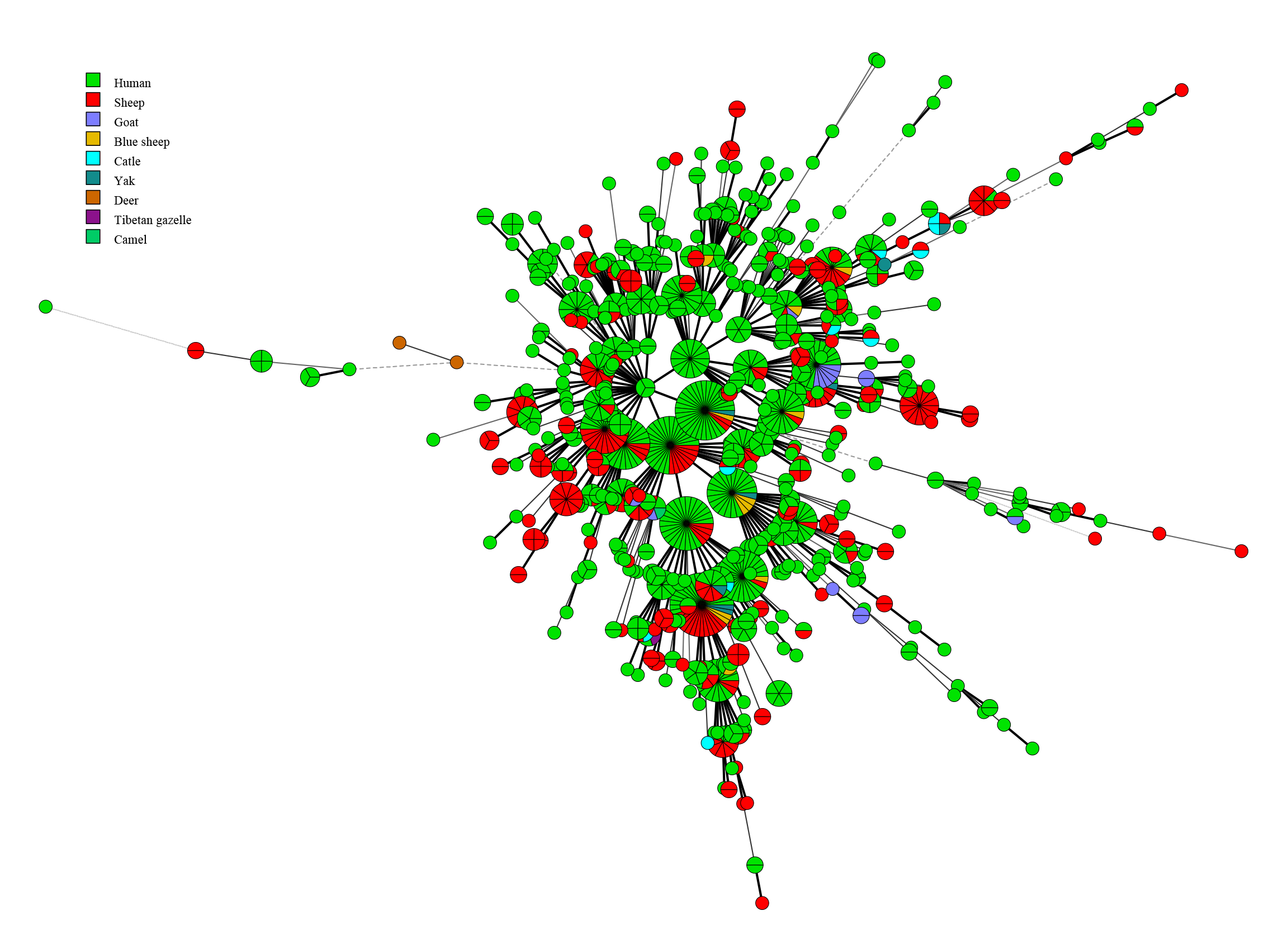

Supplement: S._Fig._8.tif [file TEMI_A_1788995_SM8492.tif]

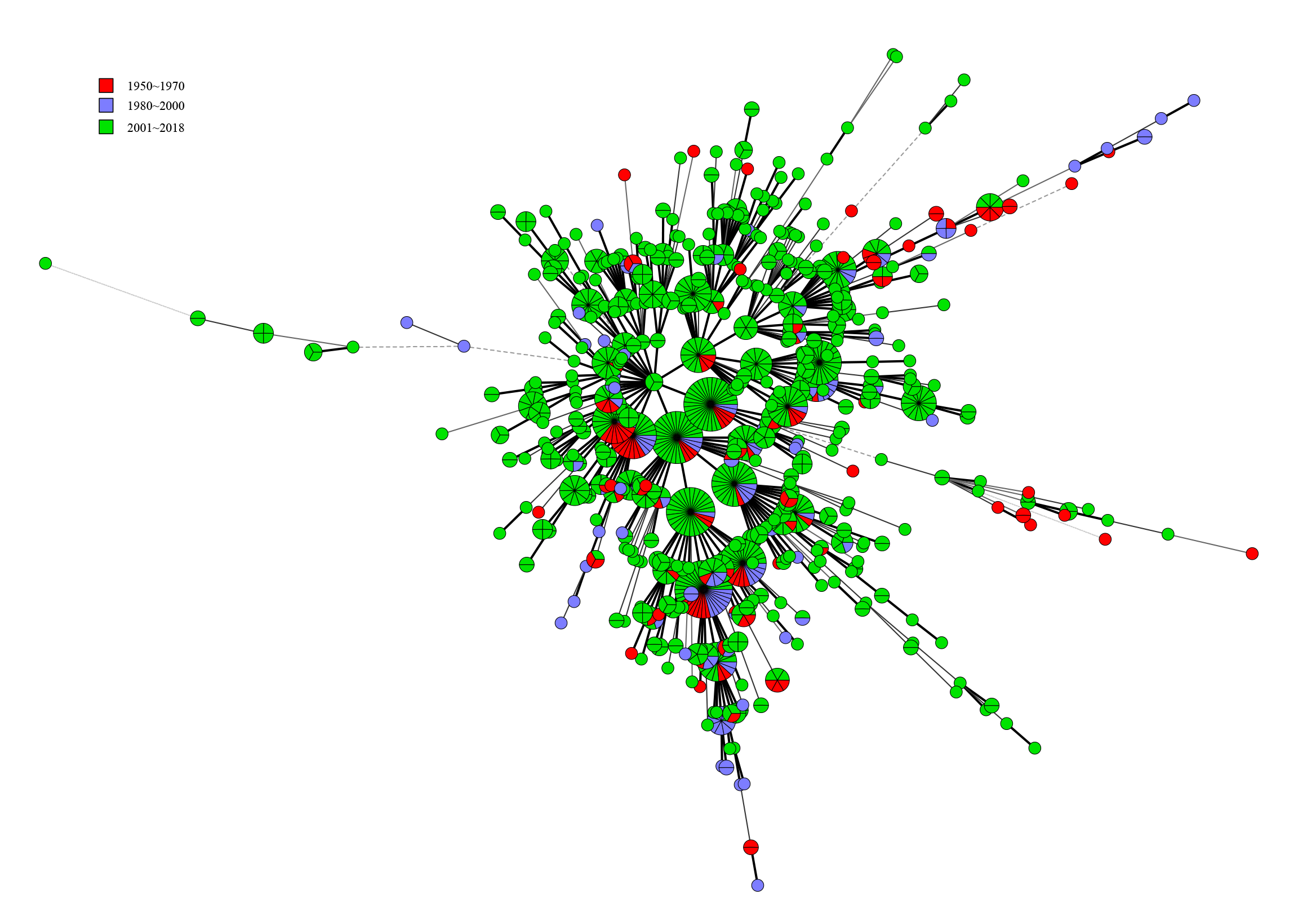

Supplement: S._Fig._7.tif [file TEMI_A_1788995_SM8491.tif]

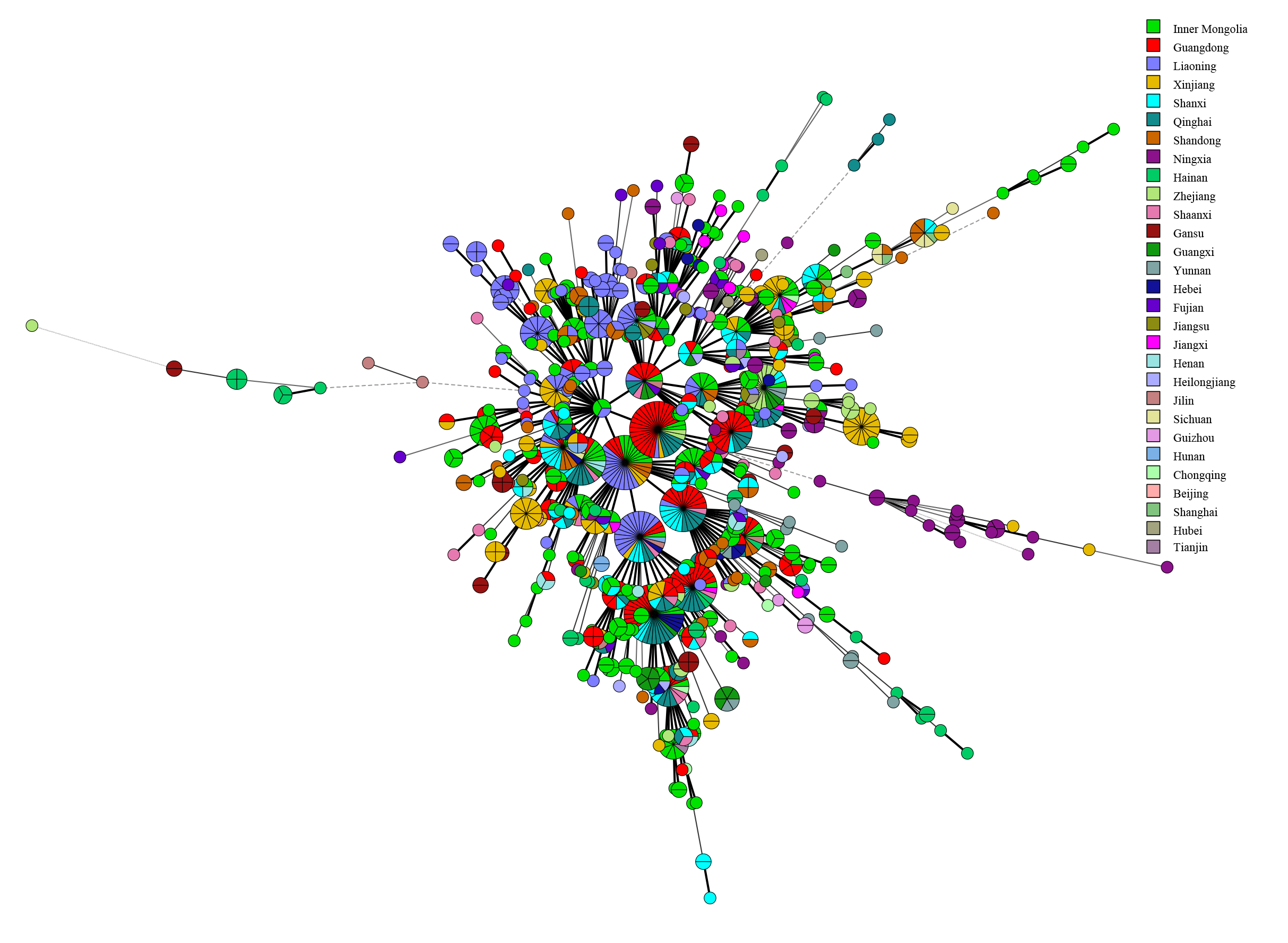

Supplement: S._Fig._6.tif [file TEMI_A_1788995_SM8490.tif]

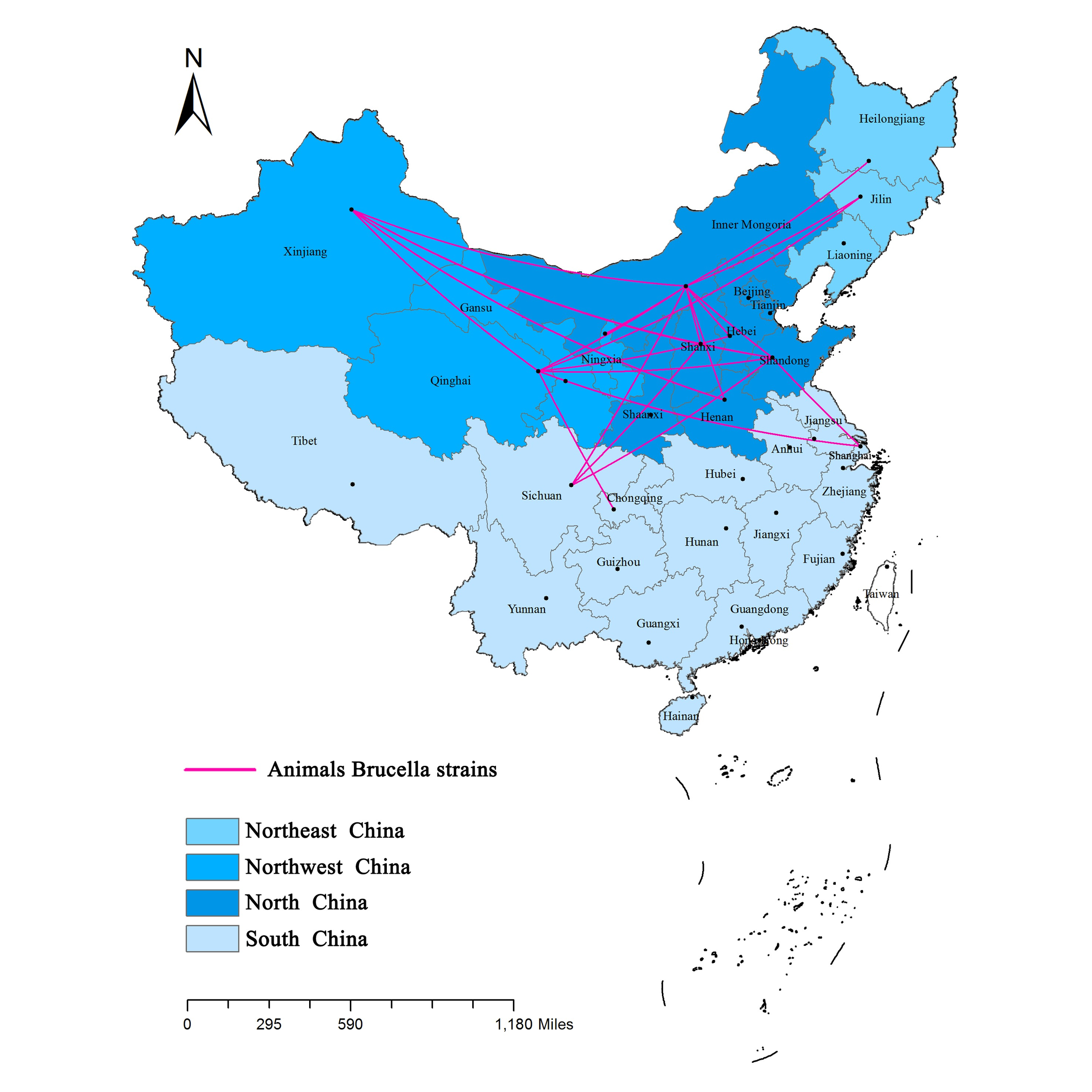

Supplement: S._Fig._5.tif [file TEMI_A_1788995_SM8489.tif]

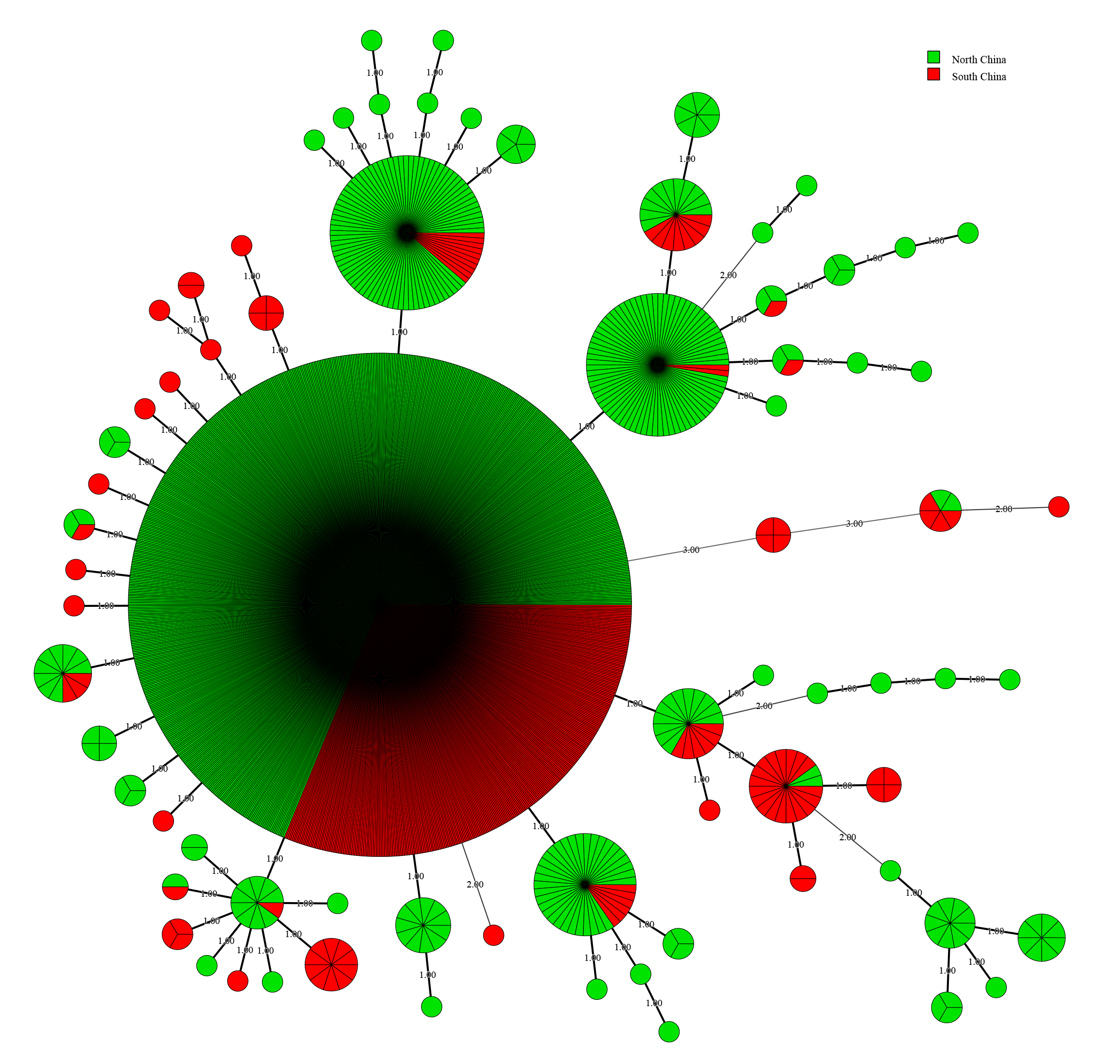

Supplement: S._Fig._4.tif [file TEMI_A_1788995_SM8488.tif]

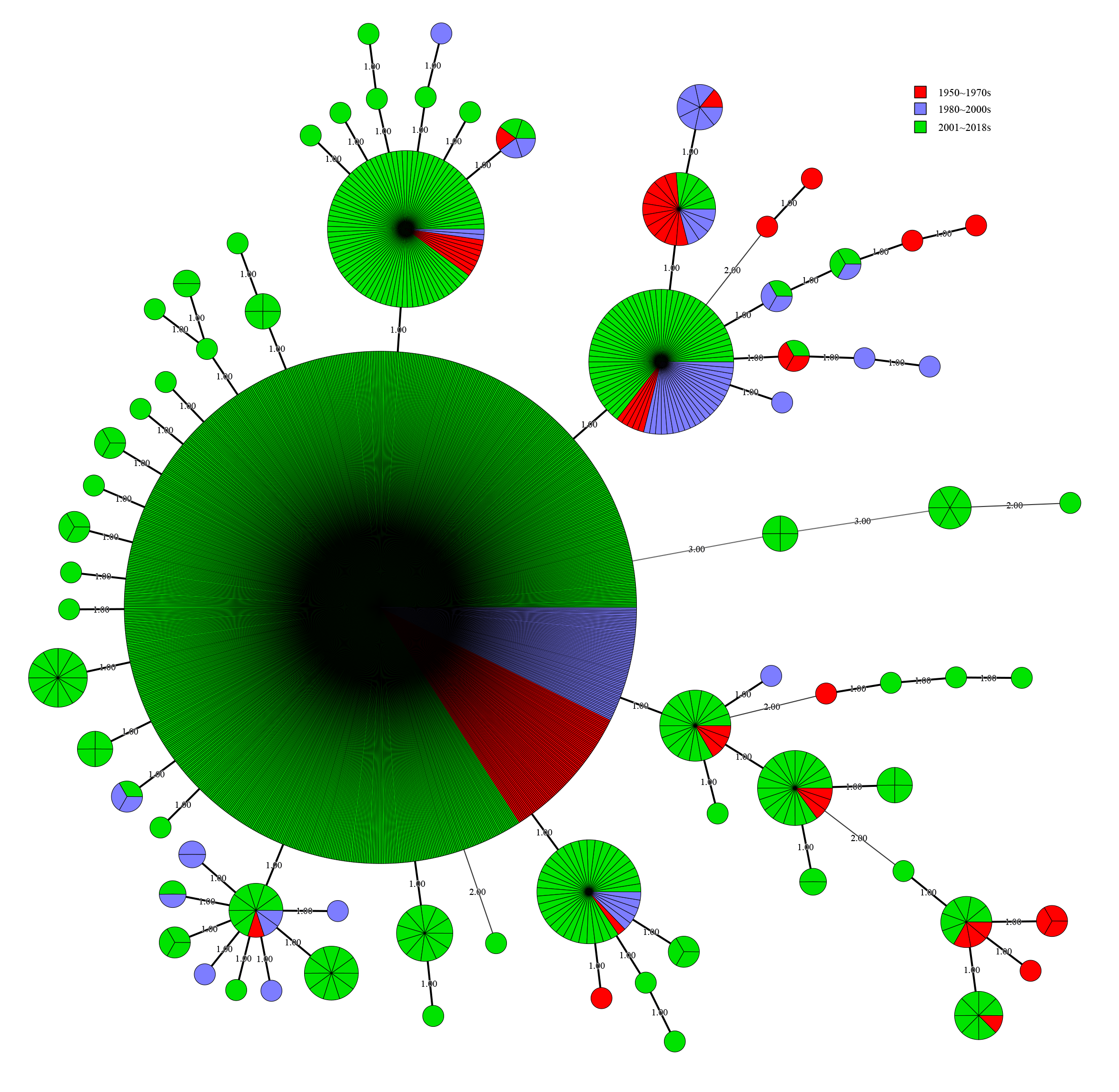

Supplement: S._Fig._3.tif [file TEMI_A_1788995_SM8487.tif]

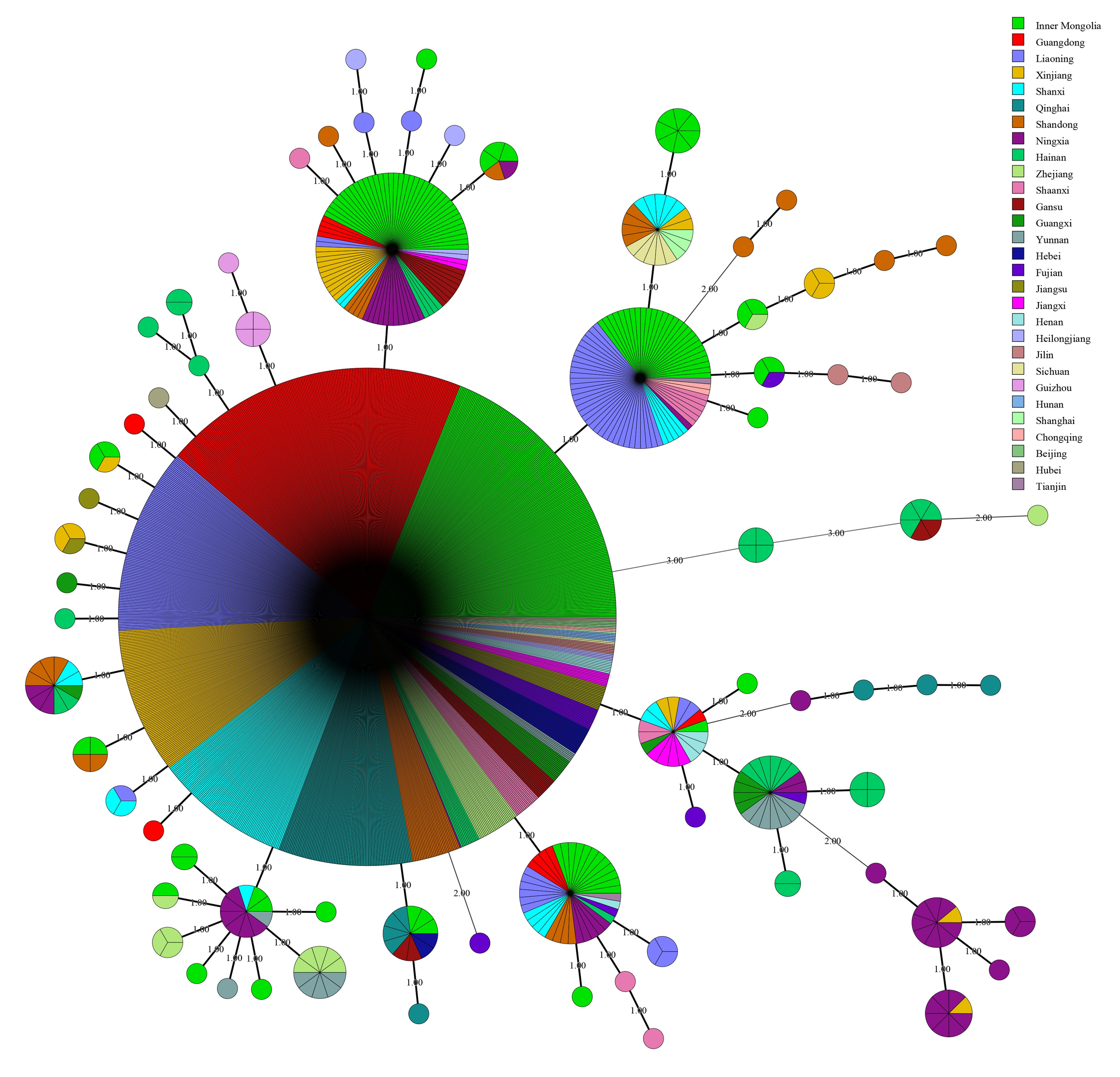

Supplement: S._Fig._2.tif [file TEMI_A_1788995_SM8486.tif]

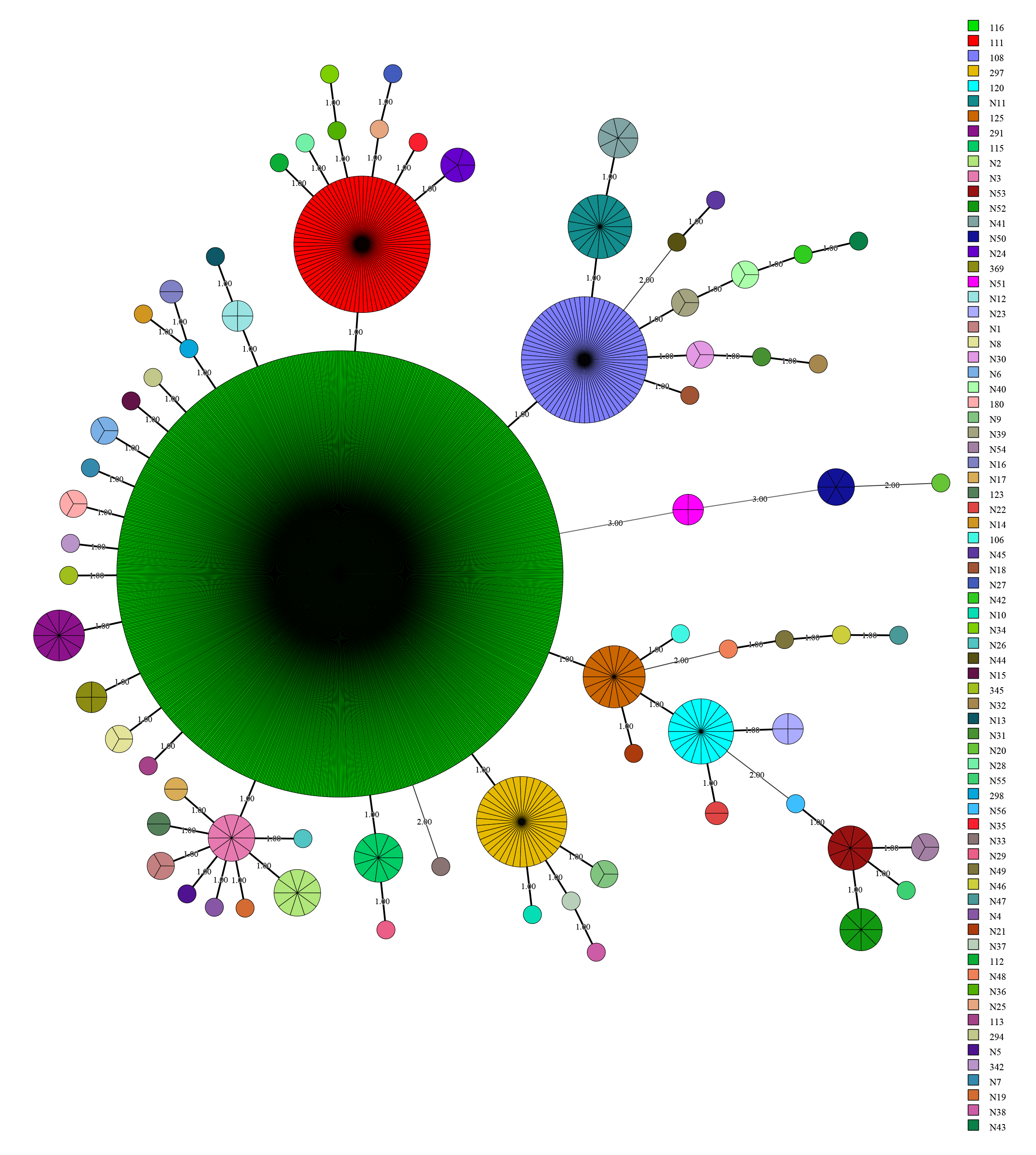

Supplement: S._Fig._1.tif [file TEMI_A_1788995_SM8485.tif]
